# MicroRNA-194-3p impacts autophagy and represses rotavirus replication via targeting silent information regulator 1

**DOI:** 10.1186/s12985-023-02175-z

**Published:** 2023-09-11

**Authors:** Haohai Huang, Dan Liao, Guanghui Zhou, Bin He, Rong Pu, Yejia Cui

**Affiliations:** 1 Department of Clinical Pharmacy, SSL Central Hospital of Dongguan, Dongguan Third People’s Hospital, Affiliated Dongguan Shilong People’s Hospital of Southern Medical University, Dongguan, Guangdong China; 2 Medical and Pharmacy Research Laboratory, SSL Central Hospital of Dongguan, Dongguan Third People’s Hospital, Affiliated Dongguan Shilong People’s Hospital of Southern Medical University, No.1, Huangzhou Xianglong Road of Shilong Town, 523326 Dongguan, Guangdong China; 3 Department of Gynaecology, SSL Central Hospital of Dongguan, Dongguan Third People’s Hospital, Affiliated Dongguan Shilong People’s Hospital of Southern Medical University, Dongguan, Guangdong China; 4 Department of Rehabilitation medicine, SSL Central Hospital of Dongguan, Dongguan Third People’s Hospital, Affiliated Dongguan Shilong People’s Hospital of Southern Medical University, Dongguan, Guangdong China; 5 Department of Clinical Laboratory, SSL Central Hospital of Dongguan, Dongguan Third People’s Hospital, Affiliated Dongguan Shilong People’s Hospital of Southern Medical University, Dongguan, Guangdong China

**Keywords:** Rotavirus, MicroRNA-194-3p, Silent information regulator 1, Viral replication, Autophagy, p53 acetylation

## Abstract

**Background:**

Rotavirus (RV) is the main cause of serious diarrhea in infants and young children worldwide. Numerous studies have demonstrated that RV use host cell mechanisms to motivate their own stabilization and multiplication by degrading, enhancing, or hijacking microRNAs (miRNAs). Therefore, exploring the molecular mechanisms by which miRNAs motivate or restrain RV replication by controlling different biological processes, including autophagy, will help to better understand the pathogenesis of RV development. This study mainly explored the effect of miR-194-3p on autophagy after RV infection and its underlying mechanism of the regulation of RV replication.

**Methods:**

Caco-2 cells were infected with RV and used to measure the expression levels of miR-194-3p and silent information regulator 1 (SIRT1). After transfection with plasmids and RV infection, viral structural proteins, RV titer, cell viability, and autophagy-linked proteins were tested. The degree of acetylation of p53 was further investigated. A RV-infected neonatal mouse model was constructed in vivo and was evaluated for diarrhea symptoms and lipid droplet formation.

**Results:**

The results showed that miR-194-3p was reduced but SIRT1 was elevated after RV infection. Elevation of miR-194-3p or repression of SIRT1 inhibited RV replication through the regulation of autophagy. The overexpression of SIRT1 reversed the effects of miR-194-3p on RV replication. The upregulation of miR-194-3p or the downregulation of SIRT1 repressed RV replication in vivo. MiR-194-3p targeted SIRT1 to decrease p53 acetylation.

**Conclusion:**

These results were used to determine the mechanism of miR-194-3p in RV replication, and identified a novel therapeutic small RNA molecule that can be used against RV.

## Background

Rotavirus (RV) is an enteric pathogen that is characterized by a non-enveloped double-stranded RNA virus belonging to the Reoviridae family, which is a major cause of severe gastroenteritis and diarrhea in young children [1]. Similar to other RNA viruses, RV relies on the host cell for protein translation and genome replication [2]. It has been reported that RV uses paracrine purinergic signaling to generate intercellular calcium waves that amplify host cell dysregulation, leading to the dysregulation of intestinal homeostasis and severe diarrhea [3]. Although the RV vaccine has been promoted in many countries and it can effectively prevent gastroenteritis caused by RV infection, currently there are no effective drugs that can cure RV infection [4]. There is abundant evidence that microRNAs (miRNAs) can affect the replication of RV viruses [5]. Therefore, exploring the molecular mechanisms by which miRNAs induce or restrain RV replication by controlling different cellular processes, will help us gain a better understanding of the pathogenesis of RV for the development of brand-new RV drugs.

MiRNAs are a class of single-stranded non-coding RNAs that can modulate gene expression post-transcriptionally by controlling mRNA stability or translation, and participate in almost all biological processes, such as cell differentiation, metabolism, and apoptosis [6]. Numerous studies have demonstrated that miRNAs key functions in complex networks of host-pathogen interactions and innate immunity [7]. Many viruses use host cell mechanisms to motivate their own stabilization and multiplication by degrading, enhancing, or hijacking miRNAs [8]. For example, miR-126 and miR-133a have been found to restrain dengue virus replication, and miR-128 is able to depress human immunodeficiency virus (HIV) replication [9–11]. A previous study found that miR-194-3p is under-expressed during RV infection [12]. However, the role and mechanism of miR-194-3p during RV replication remains to be further explored.

Autophagy is a biological process that involves catabolism in vivo that targets exogenous microorganisms and aging intracellular proteins and organelles and directs them to lysosomes for phagocytosis and degradation [13]. It has been reported that activation or repression of autophagy is induced by cells that are stimulated by exogenous pathogens, such as viruses [14]. Due to the antiviral effect of autophagy, a large number of viruses may evade and resist this process by encoding viral proteins [15]. RV infection can initiate autophagy, and can further manipulate autophagy membrane transport to transport viral endoplasmic reticulum-associated proteins to viral plasma, viral genome replication sites, and immature particle assembly sites [16]. MiRNAs modulate autophagy through signaling pathways and perform a crucial role in the treatment of various diseases [17]. Therefore, it was speculated that miRNAs can affect RV replication by modulating autophagy.

This study mainly explored the effect of miR-194-3p on autophagy after RV infection and its underlying mechanism to evaluate its function in RV replication. Through in vivo and in vitro experiments, it was found that miR-194-3p restrained RV replication by constraining autophagy by targeting silent information regulator 1 (SIRT1) to decrease p53 acetylation. This finding offers novel insights into the molecular mechanism by which miR-194-3p modulates RV replication, as well as identifies a novel target for the development of anti-RV drugs.

## Materials and methods

### Cells and viruses

Caco-2 cells (ATCC, USA) were routinely cultured in FBS (10%, Gibco), penicillin (100 U/mL), and streptomycin (100 mg/mL) containing Dulbecco’s-modified Eagle’s medium (DMEM, Gibco, USA). The wild RV strains were kindly provided by Dr. Haiyang He (Institute of Immunology, Third Military Medical University, China). RV was activated by 20 g/mL trypsin for 1 h at 37 °C and adaptively cultured with the Caco-2 cells.

### Cell transfection

MiR-194-3p mimics, inhibitors, sh/oe-SIRT1 and their negative controls (NCs) were synthesized by GenePhama (Shanghai, China). Caco-2 cells were seeded into 12-well plates until the cells covered 80% of the bottom and were transfected using a Lipofectamine 2000 system (Invitrogen, USA), according to the manufacturer’s instructions. After transfection, the cells were infected with RV and the supernatants were harvested at 12 or 24 h later.

### Expansion and titer determination of RV in the Caco-2 cells

To amplify the virus, the RV was thawed at 4 °C and 10 µg/ml ethylene diamine tetra acetic acid (EDTA)-free trypsin was added. The Caco-2 cells grown as monolayers were mixed with the RV and routinely cultured. The cytopathic effect (CPE) of the Caco-2 cells was observed under an inverted microscope until the CPE was approximately + + or +++. Next, the cells were incubated at -20 °C. After 12 h, the cells were thawed at 4 °C. The above procedure was repeated 3 times, the cell freeze-thaw solution was centrifuged, and the virus collected was packaged in sterile centrifuge tubes and stored at -80 °C. To determine the titer of the RV, the RV was first diluted in FBS-free DMEM to create a range of concentrations, including 10^1^, 10^2^, 10^3^, 10^4^, 10^5^, and 10^6^, and then reacted with Caco-2 cells at a concentration of 100 µl/well. The pathological changes of the Caco-2 cells after RV infection were observed at different times. When CPE was no longer present in the lowest dilution of the RV 96-well plate, the number of CPE wells in each dilution was recorded. The median tissue culture infectious dose of virus (TCID_50_) was measured using the Reed-Muench method.

### Immunofluorescence

The cells were fixed with 1 ml of 4% paraformaldehyde and washed with 200 µl of 0.5% TritonX 100. After blocking with 500 µL of 3% bovine serum albumin, the primary antibodies against VP6 (0.1 µg/mL, Genscript, Nanjing, China) and LC3B (1:100; ab192890; Abcam) were added to the cells and incubated. Then, a secondary antibody (goat anti-mouse; A-11,001; Thermo, Germany) was added and incubated. The slides were covered using coverslips and stained with 4,6-diamidino-2-phenylindole dihydrochloride (Sigma, USA). The samples were analyzed using a fluorescence microscope (Nikon, Tokyo, Japan). The average number of positive cells in at least 5 fields in each group was quantified.

### 3-(4, 5-dimethylthiazol-2-yl)-2, 5-diphenyltetrazolium bromide (MTT) assay

Cell viability was determined using MTT assay (Sigma, St. Louis, USA) according to the manufacturer’s protocol. In brief, Caco-2 cells were seeded in 96-well plates. Then, 20 µL of MTT (5 mg/mL) was added to each well and incubated. The culture supernatant was extracted and the purple insoluble MTT product was redissolved in 150 µL of dimethyl sulfoxide. Finally, the MTT concentration in each well was measured using a microplate reader at 550 nm.

### Proliferation

The cells were trypsinized with EDTA and was seeded into approximately 2000 cells in a six-well plate. Then, the cells were cultured for 10 to 14 days until visible colonies were formed. Thereafter, the cells were fixed with 4% paraformaldehyde, stained with 0.5% crystal violet, and counted when the visible colonies contained at least 50 cells.

### RNA isolation and real-time polymerase chain reaction (RT-PCR)

RNA was isolated from the tissues and cells according to the instructions provided by the manufacturer of the TRIzol reagent (R0016, Beyotime Biotechnology, Shanghai, China). Using 5 µg of RNA as a template, the reverse transcription reaction was performed using BeyoRT™ III First Strand cDNA Synthesis Kit (D7178L, Beyotime Biotechnology, Shanghai, China), according to the instructions. PCR was performed on a 7500 Fast Real-Time PCR System (ABI, USA) using PowerUp SYBR™ Green Master Mix (A25742, Thermo Fisher, USA). Glyceraldehyde-3-phosphate dehydrogenase (GAPDH) was used as an internal control for SIRT1 and U6 was used for miR-194-3p. The relative expression was analyzed using the 2^−ΔΔCt^ method. Primers were provided by Shanghai Gene Pharmaceutical Co., Ltd., China. The primary sequences used are presented in Table 1.

**Table 1:** Primer sequence

Genes	Orientation	Primer sequence (5’-3’)
*SIRT1*	Forward	TAGCCTTGTCAGATAAGGAAGGA
	Reverse	ACAGCTTCACAGTCAACTTTGT
*GAPDH*	Forward	CTCGCTTCGGCAGCACA
	Reverse	AACGCTTCACGAATTTGCGT
*miR-194-3p*	Forward	ACACTCCAGCTGGGCCAGTGGGGCTGCTGT
	Reverse	TGGTGTCGTGGAGTCG
*U6*	Forward	CTCGCTTCGGCAGCACA
	Reverse	AACGCTTCACGAATTTGCGT
*VP6*	Forward	CAGTGATTCTCAGGCCGAATA
	Reverse	GGCGAGTACAGACTCACAAA
*Beclin1*	Forward	CTGAGGGATGGAAGGGTCTAAGA
	Reverse	CACGGTCCAGGATCTTGAAACTC

### Western blotting analysis

The samples were lysed using a radioimmunoprecipitation assay buffer. Equal amounts of the protein extract (30 µg) were loaded onto each lane and analyzed using sodium dodecyl sulfate-polyacrylamide gel electrophoresis. Subsequently, the protein was transferred onto a polyvinylidene fluoride membrane, incubated with the primary antibodies against SIRT1 (Abcam, #ab110304, UK), Beclin1 (Cell Signaling Technology, #3495, USA), Caspase 3 (Cell Signaling Technology, #9662, USA), Cleaved Caspase 3 (Cell Signaling Technology, #9661, USA), LC3 (Cell Signaling Technology, #12,741, USA), VP6 (LifeSpan BioSciences, #LS-C93378, USA), VP7 (Creative Biolabs, #MRO-2528CQ, China), and subsequently incubated with the corresponding enzyme-labeled secondary antibody (Beyotime Institute of Biochemistry, China). Finally, protein expression was detected using an enhanced chemiluminescence kit (Millipore, USA). The protein bands were quantified with reference to GAPDH.

### The luciferase activity assay

SIRT1 wild and mutant dual-luciferase reporter plasmids were designed by GenePharma (Shanghai, China). Caco-2 cells (3 × 10^4^ cells/well) were seeded into 96-well plates and cultured. Subsequently, the cells were co-transfected with miR-194-3p (Qiagen, USA) and a luciferase reporter plasmid containing the 3′ untranslated region of SIRT1 using a Lipofectamine 2000 system (Thermo Fisher Scientific, USA). Then, the luciferase assay was performed using the Luciferase Assay System (Promega, USA), and luciferase activity was measured using a luminometer (PerkinElmer 1420 Multilabel Counter, USA).

### RNA immunoprecipitation (RIP) assay

The RIP assay was performed using a Magna RNA Binding Protein Immunoprecipitation Kit (Millipore). In brief, cell lysates were incubated with a RIP buffer coated with magnetic beads conjugated to negative immunoglobulin G (IgG) or anti-SIRT1 antibodies. Immunoprecipitated RNA was obtained through proteinase k digestion, followed by reverse transcription of RNA samples into complementary DNA for real-time quantitative PCR analysis.

### A suckling mouse model of RV diarrhea

The miR-194-3p agonist and their respective negative controls (NC) were synthesized by Ribo Bio (Guangzhou, China). Twenty-four hours before viral infection, Balb/c pups aged 4–6 days were intragastrically inoculated with 10 nmol agomir-NC, miR-194-3p agomir, DMEM, and EX527 (a potent selective SIRT1 inhibitor, 3.15 mg/kg) on the fourth day of life. Then, the mice were inoculated with 30 µL of RV by gavage. The inoculated mice were housed with dams and checked for diarrhea every 12 h by lightly palpating their abdomen. Diarrhea was evaluated (0–4 points), with no fecal excretion scoring 0 points, brown moldy stools scoring 1 point, brown soft stools scoring 2 points, yellow soft stools scoring 3 points, and yellow watery stools, and perianal fecal contamination scoring 4 points, and perianal fecal contamination scoring 4 points. Mice scoring > 2 were considered to have diarrhea. The suckling mice were euthanized at 12, 24, 48, 72, and 96 h after infection, and the intestinal tissues were collected.

### Oil red O staining

The tissue sections were fixed with 4% paraformaldehyde, stained with 60% isopropanol oil red O solution, and counterstained with Mayer’s hematoxylin. The stained specimens were observed and photographed under a light microscope.

### Statistical analysis

The statistical program SPSS 21.0 (SPSS, Inc., Chicago, IL, USA) was used to analyze the data. The values were expressed as mean ± standard deviation (SD). The t-test was used for comparison between the two groups, one-way analysis of variance (ANOVA) was used for comparison between multiple groups, and Fisher’s least significant difference t-test (LSD-t) was used for pairwise comparison after ANOVA analysis. All *P* values were two-sided with *P* < 0.05 indicating statistical significance. All experiments were performed in triplicate.

## Results

### RV infection down-regulates the expression of miR-194-3p

**Fig. 1:**
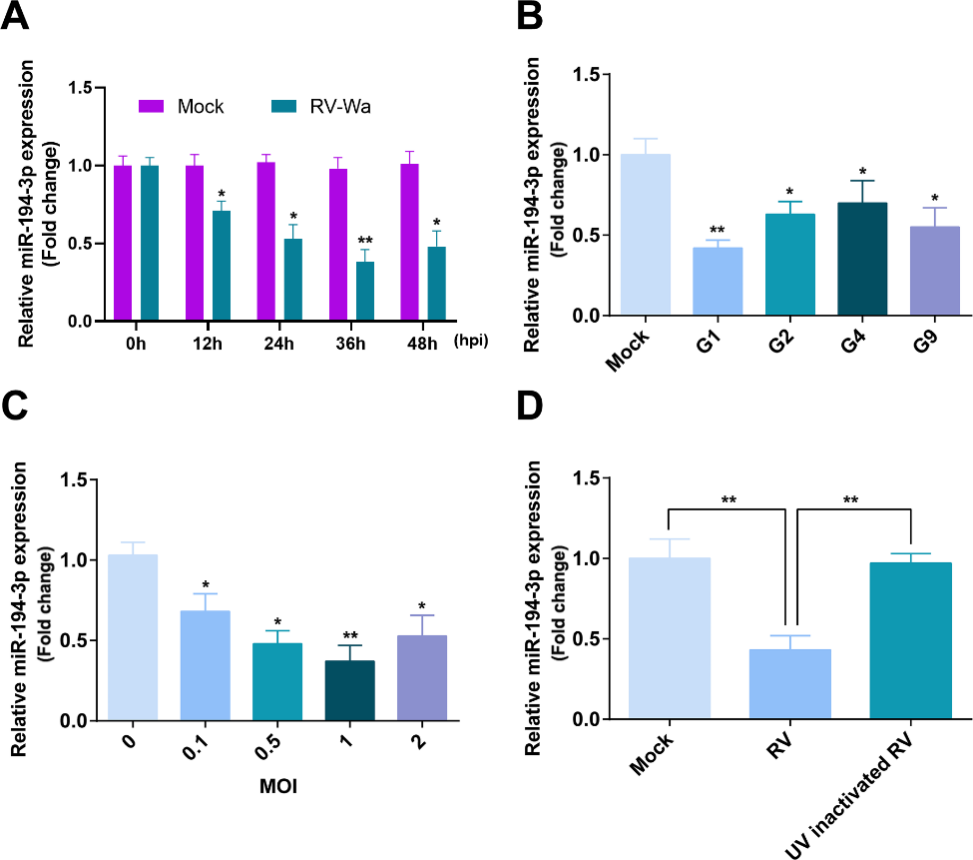
RV infection down-regulates the expression of miR-194-3p. **(A)** Caco-2 cells were infected with RV-Wa (MOI = 1) or DMEM (Mock), the expression of miR-194-3p was analyzed by using qRT-PCR at 0, 12, 24, 36 and 48 hpi. **(B)** Caco-2 cells were infected with other genotypes of RV, such as G1 (Wa), G2 (S2), G4 (Gottfried), G9 (ZTR-18), the expressions of miR-194-3p was analyzed by using qRT-PCR at 36 hpi. **(C)** Caco-2 cells were infected with different MOI (0.1, 0.5, 1, 2) of RV-Wa, the expressions of miR-194-3p was analyzed by using qRT-PCR at 36 hpi. **(D)** The expressions of miR-194-3p was measured by qRT-PCR in UV-inactivated RV-Wa infected Caco-2 cells at 36 hpi. The fold change in the transcript was calculated by normalizing the relative gene expression to U6. The data are presented as the mean ± SD. * *P* < 0.05; ** *P* < 0.01

To study the expression level of miR-194-3p during RV infection, total RNA was extracted from Caco-2 cells after infection with RV-Wa (MOI = 1) at 12, 24, 36 and 48 h post infection (hpi). The qRT-PCR results showed that miR-194-3p expression was significantly downregulated and reached a minimum value at 36 hpi (Fig. 1A). Meanwhile, miR-194-3p expression was downregulated in the Caco-2 cells infected with different RV strains (MOI = 1) at 36 hpi, covering G1 (Wa), G2 (S2), G4 (Gottfried), and G9 (ZTR-18), and was most pronounced in cells infected with the G1 strain (Fig. 1B). Therefore, RV-Wa was selected to infect the Caco-2 cells with different MOI (0.1, 0.5, 1, and 2) at 36 hpi. It was found that RV infection repressed miR-194-3p expression in the Caco-2 cells in an MOI-dependent manner (Fig. 1C). However, when the Caco-2 cells were infected with UV-inactivated RV at the same MOI at 36 hpi, miR-194-3p expression did not change, suggesting that RV specifically modulates miR-194-3p expression (Fig. 1D). The above results indicated that miR-194-3p expression is downregulated during RV infection.

### MiR-194-3p represses RV replication and autophagy during RV infection

**Fig. 2:**
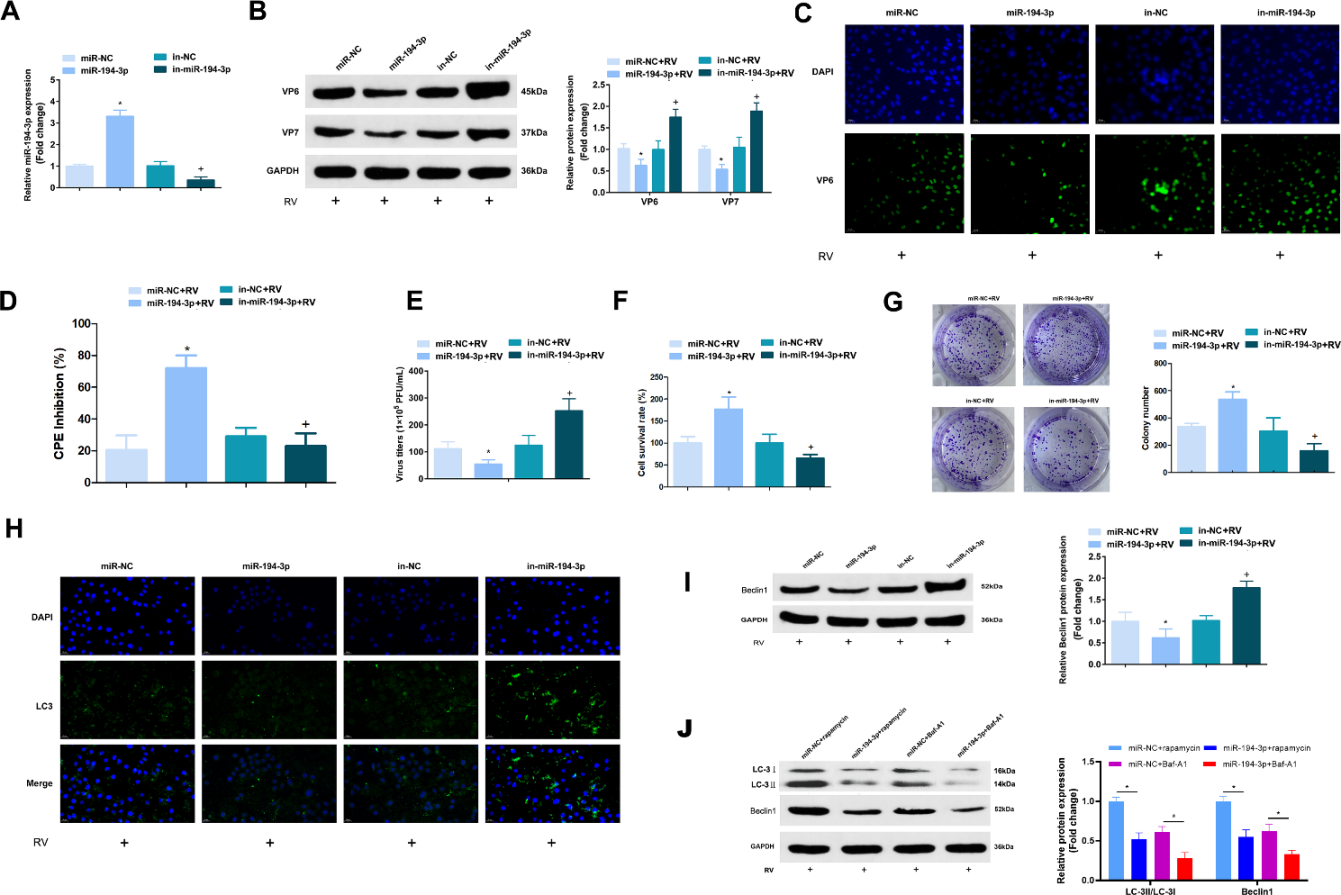
MiR-194-3p represses RV replication and autophagy during RV infection. **(A)** Caco-2 cells were transfected with negative control (NC) oligonucleotides, miR-194-3p mimics or inhibitors for 36 h, and the expression of miR-194-3p was determined by qRT-PCR. The fold change was calculated after normalized with U6. **(B)** Caco-2 cells transfected with NC oligonucleotides, miR-194-3p mimics or inhibitors were infected with RV-Wa (MOI = 1) for 24 h, the expressions of viral structural proteins VP6 and VP7 were determined by western blot. **(C)** The expressions of VP6 was determined by Immunofluorescence analysis. **(D)** The antiviral activity were determined by using CPE inhibition assays. **(E)** The infectious virus titers in cell supernatants were evaluated by using plaque assays. **F/G.** The survival rate of Caco-2 cells were assessed by MTT and plate cloning assays. **H.** The expressions of LC3 was determined by Immunofluorescence analysis. **I.** The expressions of autophagy-linked protein Beclin1 was detected by western blot. **J.** Caco-2 cells transfected with NC oligonucleotides, miR-194-3p mimics or miR-NC were infected with RV-Wa (MOI = 1), and then treated with rapamycin (100 nM) or bafilomycin A1 (Baf-A1, 100 nM) for 24 h, the cells were then harvested for detection. The expression of Beclin1 and LC3 were determined by western blot. The fold change was calculated after normalized with GAPDH. The data are presented as the mean ± SD. * vs. the miR-NC, *P* < 0.05; + vs. the in-NC, *P* < 0.05

To further explore the role of miR-194-3p during RV replication, miR-194-3p mimics and inhibitors were applied to manipulate miR-194-3p expression in the Caco-2 cells (Fig. 2A). To assess the functional consequences of RV replication and host cell resistance after altering miR-194-3p, the Caco-2 cells transfected with miR-194-3p mimics, inhibitors or their scrambled controls were infected with RV-Wa (MOI = 1) for 24 h. RV structural proteins, VP6 and VP7, were assessed using western blotting analysis. It was found that elevated miR-194-3p expression reduced VP6 and VP7 expression levels, whereas the repression of miR-194-3p expression elevated VP6 and VP7 expression levels (Fig. 2B). Meanwhile, enhancing miR-194-3p expression reduced the intensity of green fluorescence of anti-RV, while the depression of miR-194-3p elevated the intensity of green fluorescence of VP6 (Fig. 2C). Moreover, our results indicate that enhanced miR-194-3p expression caused the inhibition of viral replication of RV-Wa in the Caco-2 cells (Fig. 2D). It was subsequently discovered that during RV amplification in host cells, the transfection of miR-194-3p mimics was found to reduce RV titers, whereas the miR-194-3p inhibitor elevated the RV titers (Fig. 2E). Next, to further confirm the effect of miR-194-3p on RV, the viability of the host cells was assessed, and it was found that miR-194-3p mimics improved cell viability, while the miR-194-3p inhibitor reduced cell viability (Fig. 2F). Meanwhile, the results of the 0.1% crystal violet staining of the live cells also manifested that enhanced miR-194-3p expression in RV infection reduced the death of host cells (Fig. 2G).

Autophagy is a major modulator of RV infection, and RV motivates the formation of autophagosomes for proper viral replication [18]. By detecting autophagy-linked proteins, it was found that, miR-194-3p overexpression decreases LC3 and Beclin1 expression levels in RV-infected cells, while repressing miR-194-3p elevated LC3 and Beclin1 expression levels (Fig. 2H, I). Moreover, Caco-2 cells transfected with miR-194-3p mimics or miR-NC were infected with RV-Wa (MOI = 1), and then treated with rapamycin (an autophagy inducer, 100 nM) or bafilomycin A1 (Baf-A1, an inhibitor of autophagy, 100 nM) for 24 h. The expression of Beclin1 and LC3 after rapamycin treatment were inhibited by miR-194-3p overexpression (Fig. 2J). Together, these results suggest that miR-194-3p may repress RV replication and autophagy during RV infection.

### MiR-194-3p regulates RV replication by targeting SIRT1

**Fig. 3:**
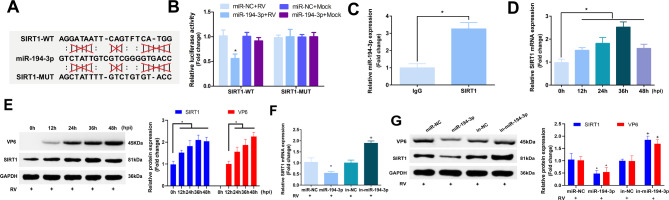
MiR-194-3p regulates RV replication by targeting SIRT1. **(A)** Bioinformatics website RNA22 was used to detect the binding site of SIRT1 and miR-194-3p. **(B)** Caco-2 cells transfected with miR-194-3p mimics or NC oligonucleotides were infected with RV-Wa (MOI = 1) or DMEM (Mock) for 24 h. The interaction between miR-194-3p and SIRT1 was verified by dual-luciferase reporter assay. **(C)** Caco-2 cells were infected with RV-Wa at an MOI of 1 for 24 h, the interaction between miR-194-3p and SIRT1 was detected by RIP analysis. **D/E.** Caco-2 cells were infected with RV-Wa at an MOI of 1, the culture supernatant and cell lysates were collected at indicated time points (0, 12, 24, 36, 48 hpi), the mRNA and proteins expression of SIRT1 and VP6 were determined by qRT-PCR and western blot, respectively. **F/G.** Caco-2 cells transfected with NC oligonucleotides, miR-194-3p mimics or inhibitors were infected with RV-Wa (MOI = 1) for 24 h, the mRNA and proteins expression of SIRT1 and VP6 were determined by qRT-PCR and western blot, respectively. The fold change was calculated after normalized with GAPDH. The data are presented as the mean ± SD. * vs. the miR-NC, *P* < 0.05; + vs. the in-NC, *P* < 0.05

Bioinformatics website RNA22 (https://cm.jefferson.edu/rna22/) showed that SIRT1 has a binding site for miR-194-3p (Fig. 3A). Site-directed mutagenesis was applied to generate mutated SIRT1 sequences (Named SIRT1-MUT). Meanwhile, miR-194-3p mimics decreased the luciferase activity of SIRT1-wt during RV infection, but not SIRT1-MUT (Fig. 3B). The RIP results manifested that miR-194-3p could be precipitated by the SIRT1 antibody during RV infection, but not by IgG (Fig. 3C). Furthermore, when the Caco-2 cells were infected with RV-Wa at an MOI of 1, the expression of SIRT1 was significantly upregulated and reached a maximum value at 36 hpi (Fig. 3D, E). The Caco-2 cells transfected with miR-194-3p mimics, inhibitors or their scrambled controls were infected with the RV-Wa strain (MOI = 1) for 24 h, and the expression of SIRT1 at mRNA and protein level were determined using qRT-PCR and western blotting analysis, respectively. The result showed miR-194-3p significantly inhibited the expression of SIRT1 (Fig. 3F, G). The results of the above-mentioned experiments indicate that miR-194-3p targets SIRT1.

### SIRT1 regulates autophagy and RV replication during RV infection

**Fig. 4:**
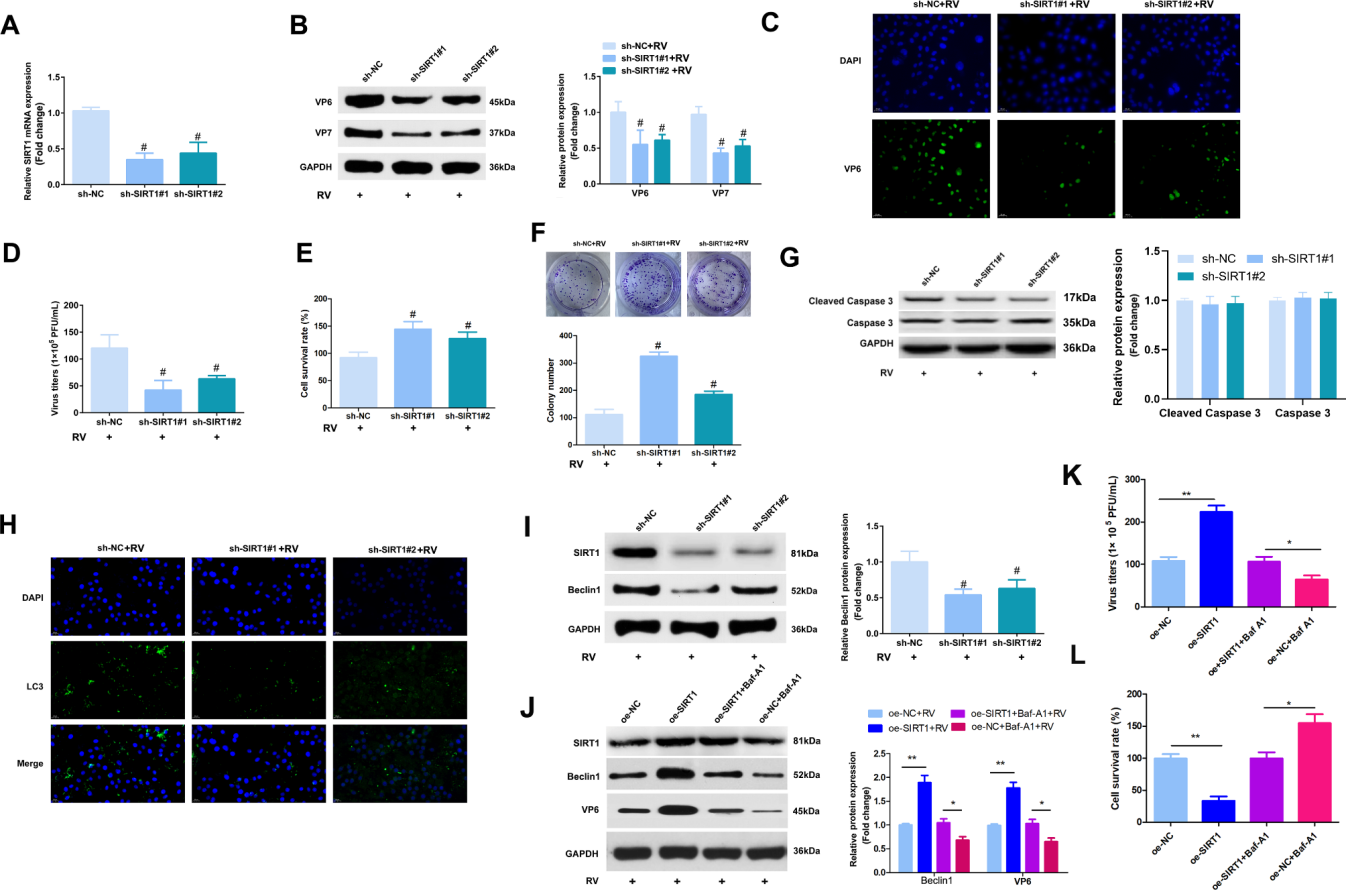
Inhibition of SIRT1 expression restrains autophagy and RV replication. **(A)** The mRNA expression of SIRT1 in Caco-2 sh-SIRT1 cells was detected by qRT-PCR. **(B)** Caco-2 cells transfected with sh-NC, sh-SIRT1#1 or sh-SIRT1#2 were infected with RV-Wa (MOI = 1) for 24 h, and the cells were then harvested for detection. The expression of RV structural proteins VP6 and VP7 were detected by western blot. **(C)** The expression of VP6 was detected by Immunofluorescence analysis. **(D)** The RV titers was detected by using plaque assays. **E/F.** The survival of Caco-2 cells were assessed by MTT and plate cloning. **G.** The expression of Cleaved caspase 3 and Caspase 3 were detected by western blot. **H.** The expression of autophagy-linked protein LC3 was detected by Immunofluorescence. **I.** The Beclin1 and SIRT1 were determined by western blot. **J.** Caco-2 cells transfected with oe-SIRT1 or oe-NC were infected with RV-Wa (MOI = 1), and then treated with DMEM or bafilomycin A1 (Baf-A1) for 24 h, the cells were then harvested for detection. The expressions of Beclin1, SIRT1 and VP6 were determined by western blot. **K.** The infectious virus titers in cell supernatants were evaluated by using plaque assays. **L.** The survival of Caco-2 cells were assessed by MTT. The fold change was calculated after normalized with GAPDH. The data are presented as the mean ± SD. # vs. the sh-NC, *P* < 0.05

To explore whether SIRT1 performs a similar function as miR-194-3p in RV replication, the expression of SIRT1 in Caco-2 cells was interfered by short hairpin RNA (shRNA). To avoid off-target effects, two different shRNAs that target SIRT1 (sh-SIRT1#1 and sh-SIRT1#2) were applied. Both SIRT1 shRNAs could effectively downregulate SIRT1, but sh-SIRT1#1 was more effective (Fig. 4A). The Caco-2 cells were infected with RV-Wa at an MOI of 1 for 24 h, and the results showed that repression of SIRT1 repressed the RV structural proteins, VP6 and VP7 (Fig. 4B, C), reduced the RV titer (Fig. 4D), and elevated the viability of the Caco-2 cells (Fig. 4E, F). We further examined the effect of interference of SIRT1 expression on cell apoptosis. The result showed that inhibition of SIRT1 expression did not affect apoptosis during RV infection (Fig. 4G). Moreover, it was also found that SIRT1 regulates autophagy during RV infection, thereby affecting the viral replication process (Fig. 4H, I, J). However, the overexpression of SIRT1 promoted RV replication (Fig. 4K) and reduced the viability of the Caco-2 cells (Fig. 4L).

### Over-expression of SIRT1 reverses the depressive effect of mir-194-3p on RV replication

**Fig. 5:**
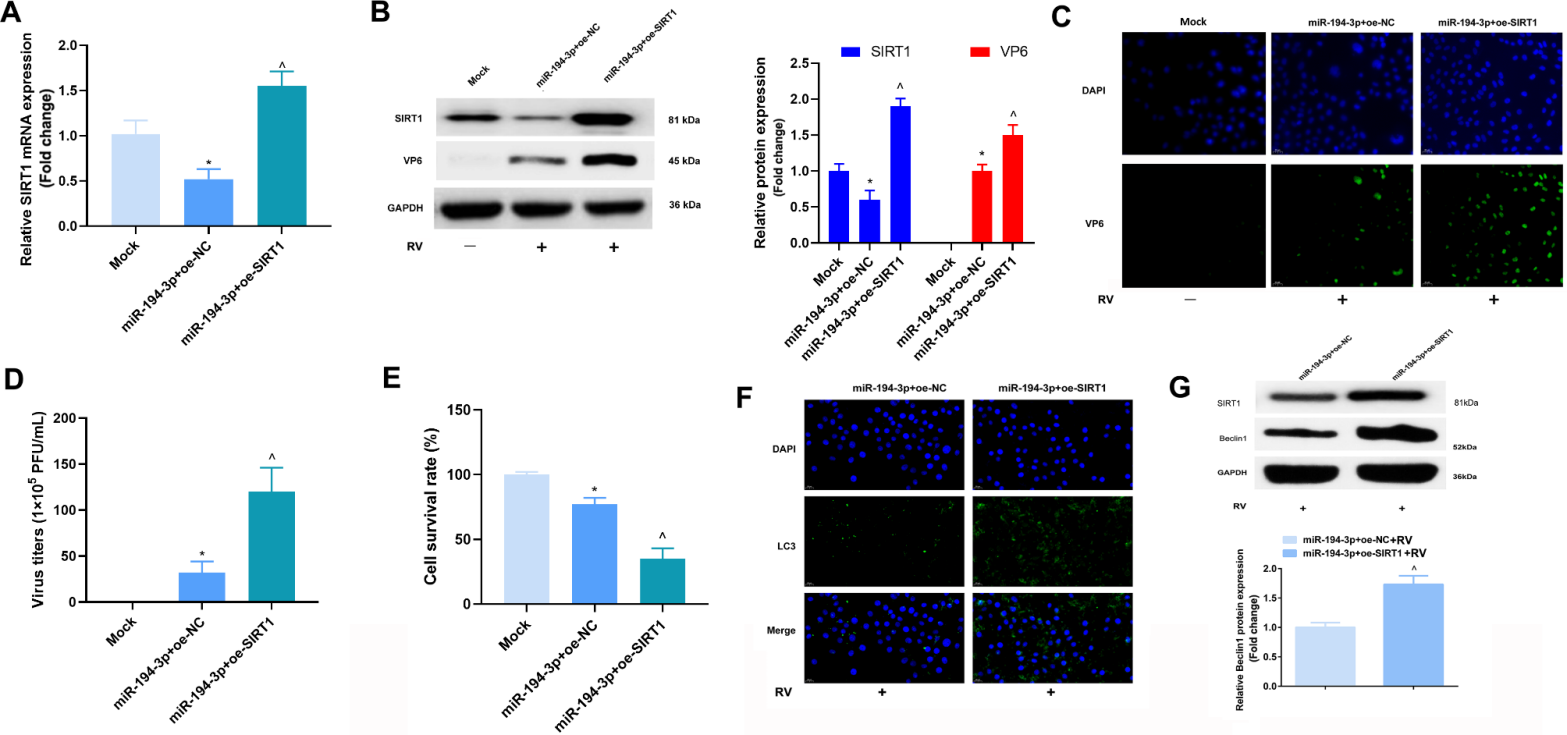
Over-expression of SIRT1 reverses the depressive effect of miR-194-3p on RV replication. **(A)** Caco-2 cells transfected with miR-194-3p mimics were than transfected with oe-NC or oe-SIRT1. The expression of SIRT1 was detected by qRT-PCR. (B) Caco-2 cells transfected with miR-194-3p mimics + oe-NC or miR-194-3p mimics + oe-SIRT1 were infected with RV-Wa (MOI = 1) for 24 h, and the cells were then harvested for detection. The expression of VP6 and SIRT1 were detected by western blot. **(C)** The expression of VP6 was detected by Immunofluorescence analysis. **(D)** The RV titers was detected by using plaque assays. **E/F.** The survival of Caco-2 cells were assessed by MTT and plate cloning. **G.** The expression of LC3 was detected by Immunofluorescence. **H.** The expression of SIRT1 and Beclin1 were determined by western blot. The fold change was calculated after normalized with GAPDH. The data are presented as the mean ± SD. *vs. the Mock, *P* < 0.05; ^ vs. the miR-194-3p + oe-NC, *P* < 0.05

Next, the mechanism of action of the miR-194-3p/SIRT1 axis in RV replication was further elucidated. Oe-SIRT1 was transfected into Caco-2 cells with miR-194-3p mimics, resulting in an increase in the expression of SIRT1 (Fig. 5A). Subsequently, the Caco-2 cells transfected with miR-194-3p mimics + oe-NC or miR-194-3p mimics + oe-SIRT1 were infected with RV-Wa (MOI = 1) for 24 h. The results showed that the enhancement of SIRT1 reversed the effects of miR-194-3p on the VP6 protein (Fig. 5B, C**)**, RV titers (Fig. 5D), cell viability (Fig. 5E), and autophagy (Fig. 5F, G). The above-mentioned results indicated that the overexpression of SIRT1 reversed the depressive effect of miR-194-3p on RV replication.

### Upregulating mir-194-3p or depressing SIRT1 restrained RV replication in vivo

**Fig. 6:**
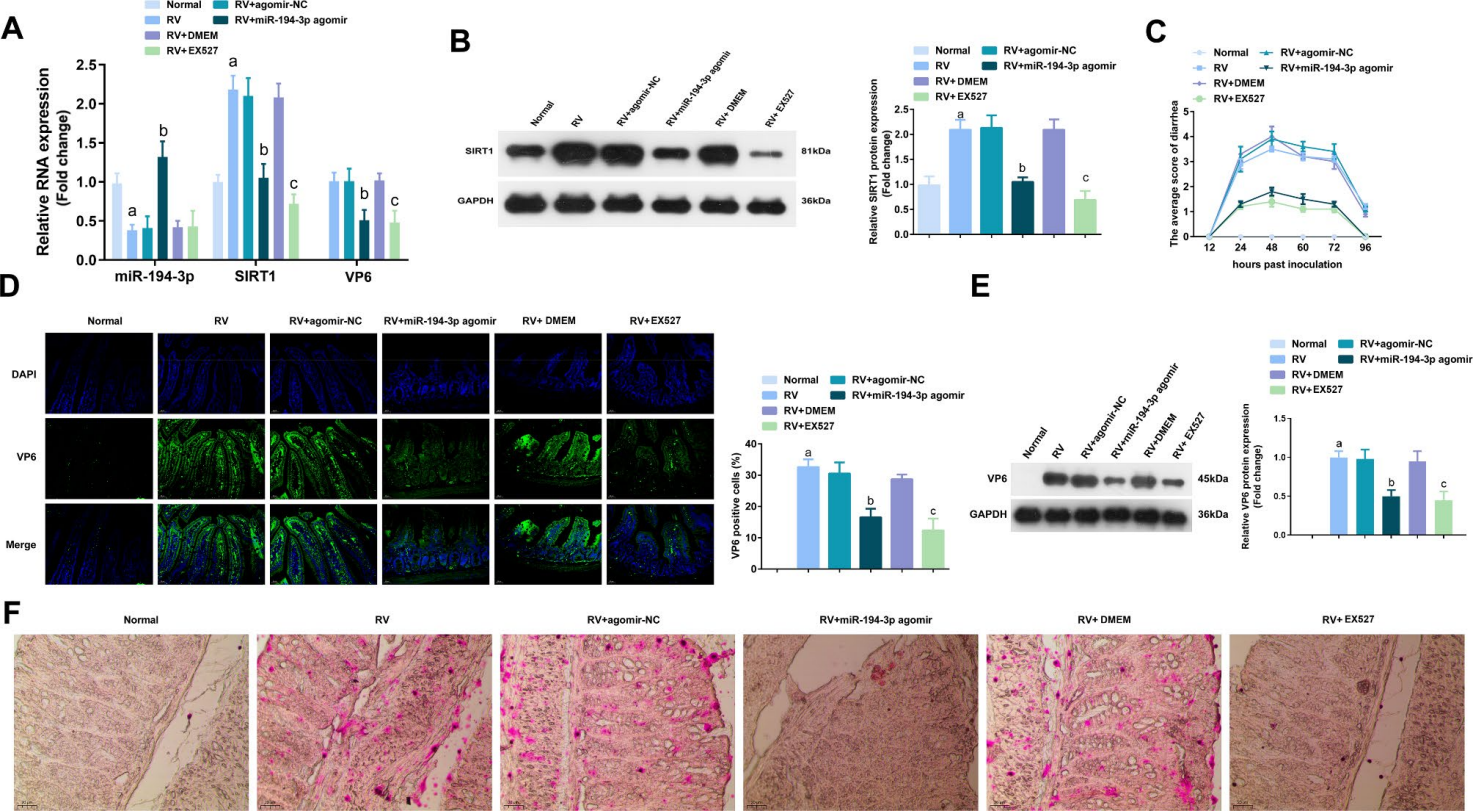
Upregulating miR-194-3p or depressing SIRT1 restrained RV replication *in vivo*. **(A)** The suckling mice were respectively treated with 10 nmol agomir-NC, miR-194-3p agomir, DMEM, and EX527 (3.15 mg/kg) by oral gavages and then inoculated with 30 µL RV through oral gavages, and the intestinal tissue were harvested for detection at 96 hpi. The expression of miR-194-3p, VP6 and SIRT1 were detected by qRT-PCR. **(B)** The protein expression of SIRT1 in suckling mice were detected by western blot. **(C)** The mean diarrheal score for each group at days form 12–96 hpi. **D/E.** The expression of VP6 in intestinal tissue was determined by immunofluorescence analysis and western blot, respectively. **F.** The lipid droplet formation in intestinal tissue was detected by Oil red O staining. The fold change was calculated after normalized with GAPDH. The data are presented as the mean ± SD. a vs. the Normal, *P* < 0.05. b vs. the agomir-NC, *P* < 0.05. c vs. the DMEM, *P* < 0.05

To further investigate the antiviral effect of the miR-194-3p/SIRT1 axis, a RV-infected neonatal mouse model was established. The suckling mice were treated with 10 nmol agomir-NC, miR-194-3p agomir, DMEM, and EX527 through gavage and then inoculated with RV through oral gavage. The results showed that the expression level of SIRT1 decreased after miR-194-3p agomir or EX527 intervention. **(**Fig. 6A, B). The symptoms of diarrhea were scored using the Boshuizen scoring standard [19]. The results showed that 24 h after RV infection, mice treated with agomir-NC or DMEM showed severe diarrhea symptoms (both 4 points) and began to recover at 96 h after infection, while no obvious symptoms of diarrhea were observed in mice treated with miR-194-3p agomir or EX527 (Fig. 6C). After the mice were euthanized, intestinal tissue was collected. RV replication in the intestinal tissue was analyzed through immunofluorescence analysis and western blotting, respectively. The results showed that the upregulation of miR-194-3p or repression of SIRT1 reduced the positive expression of VP6 (Fig. 6D, E). After RV infection, there was an increased in the number of lipid droplets formed and aggregated in the intestinal tissue of the infected mice. However, lipid droplet formation and aggregation were attenuated in mice treated with miR-194-3p agomir or EX527 (Fig. 6F). In brief, elevation of miR-194-3p or repression of SIRT1 inhibited RV replication in vivo.

#### MiR-194-3p depresses p53 acetylation by modulating SIRT1 during RV infection

**Fig. 7:**
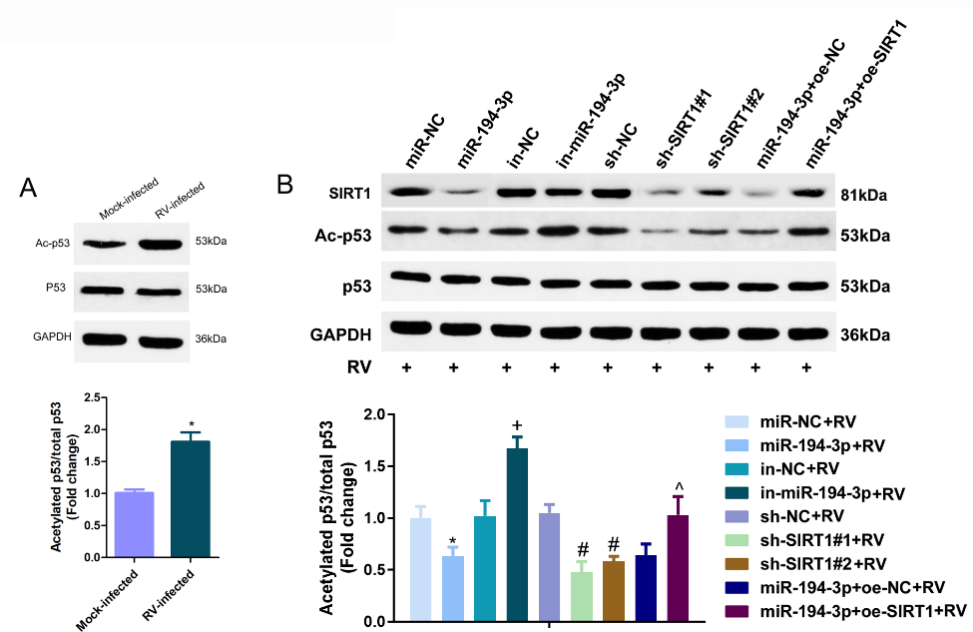
MiR-194-3p depresses p53 acetylation by modulating SIRT1 during RV infection. **(A)** Caco-2 cells were infected with RV-Wa (MOI = 1) or DMEM (Mock) for12 hpi, followed by western blot with anti-p53 and anti-Ac-p53 antibody. **(B)** Caco-2 cells transfected with miR-194-3p mimics or inhibitors or sh-NC, sh-SIRT1#1 or sh-SIRT1#2 or miR-194-3p mimics + oe-NC or miR-194-3p mimics + oe-SIRT1 were infected with RV-Wa (MOI = 1) for 12 hpi, the cells were then harvested for detection. Western blot were used to detect the expression of SIRT1, p53 and acetylation of p53 in each group of cells. The fold change was calculated after normalized with GAPDH. The data are presented as the mean ± SD. * vs. the miR-NC, *P* < 0.05. + vs. the in-NC, *P* < 0.05. # vs. the sh-NC, *P* < 0.05; ^ vs. the miR-194-3p + oe-NC, *P* < 0.05

p53 acetylation is a key event in p53-mediated antiviral responses [20]. Western blotting analysis was performed on Caco-2 cells infected with RV-Wa (MOI = 1) or DMEM (Mock) for 12 hpi. Compared with the Mock-infected group, levels of p53 acetylation were significantly elevated in RV-infected Caco-2 cells during early infection (Fig. 7A). SIRT1, a histone deacetylase, has been reported to induce the acetylation of p53 [21]. Therefore, it was further investigated whether SIRT1 induced p53 acetylation in RV-infected Caco-2 cells at 12 hpi. Detection of p53 and Ac-p53 in each group of cells showed that the elevation in miR-194-3p expression or depression in SIRT1 constrained the acetylation of p53, while a reduction in miR-194-3p induced the acetylation of p53. Overall, up-regulation of SIRT1 reversed the depression of p53 acetylation mediated by the upregulation of miR-194-3p (Fig. 7B).

## Discussion

RV infection remains as the main cause of acute gastroenteritis in young children worldwide. Despite four oral, live, attenuated anti-RV vaccines are in action, there is no substantial decrease in the prevalence of Rotavirus infection. It is still one of the significant causes of morbidity and mortality in young children [22]. A possible reason could be that these vaccines fail to provide complete cross-protection which may lead to a selective increase of the less prevalent strains, unusual genotypes or the unusual combinations of common genotypes. This scenario leads to the need to develop antiviral compounds against RV infection [23, 24]. The host-mediated factors that lead to viral infections have been an important objective for the study of the development of antiviral drugs as anti-RV therapies. Gene silencing, especially RNA interference, has been reported to offer a new alternative method for drug development [25]. Gene silencing can be conducted through mRNA degradation or inhibition, which can be performed using small interfering RNAs, short hairpin RNAs, and miRNAs [26]. It has been proven that miRNAs can exert pro-viral and antiviral properties during the pathogenesis of RV. On the one hand, miRNAs that induce RV pathogenesis are able to target cellular components to evade host antiviral strategies, while on the other hand, anti-RV-characterized miRNAs lose their ability during infection [27]. Therefore, exploring the molecular mechanisms by which miRNAs modulate RV replication is crucial for the development of new antiviral drugs. In this study, it was found that miR-194-3p expression was downregulated during RV infection. MiR-194-3p repressed RV replication by targeting SIRT1 to depress p53 acetylation to slow down autophagy.

The ability of miRNAs to affect RV replication has long been demonstrated. For example, miR-7 inhibits RV replication by targeting viral NSP5 [5]. MiR-4301 in host cells induce RV replication by targeting PPP1R3D [28]. Moreover, miRNAs play a significant role in the regulation of autophagy in viral infections [29, 30]. MiR-30a-5p can inhibit DEV replication through the decrease of autophagy by targeting Beclin-1 [29]. Ectopic expression of let-7 g and the knockdown of miR-99b result in the inhibition of autophagy, leading to a reduction in RV replication [30]. RV-encoded virus-like small RNAs can activate autophagy in host cells [31]. Autophagy has been reported to facilitate RV infection through NSP4-mediated activation of CaMKK-β and the AMPK-dependent signaling pathway [32, 33]. Previous studies have shown that miR-194-3p is aberrantly expressed in various cancers, such as hepatocellular carcinoma, breast cancer, and nasopharyngeal carcinoma [34–36]. MiR-194-3p is dysregulated in the serum of patients with chronic hepatitis B. Meanwhile, miR-194-3p expression has been reported to be downregulated during RV infection [12]. Although the function of miR-194-3p in cancer is definite, its role in the diseases caused by viral infection is not yet certain. Our present study found that miR-194-3p expression was downregulated during RV infection both in vitro and in vivo. Elevated miR-194-3p expression decreases VP6 and VP7 production, which obviously reduced the intensity of anti-RV green fluorescence and RV titers, elevated host cell survival, and repressed host cell death. Interestingly, it was further found that miR-194-3p could affect RV replication by affecting the autophagy of host cells. Elevated miR-194-3p expression downregulates the expression of autophagy-linked proteins (LC3 and Beclin1), whereas the downregulation of miR-194-3p exerts an opposite effect. Meanwhile, in vivo experimental results showed that elevation of miR-194-3p expression inhibited RV replication in suckling mice with RV diarrhea. As far as we are aware, this study for the first time demonstrates the role of miR-194-3p expression in RV replication and the association between host cell autophagy and RV replication. Many studies have clarified that miRNAs are involved in various biological processes by targeting downstream genes. Therefore, in this study, the downstream targets of miR-194-3p were further explored, which confirmed that miR-194-3p targets SIRT1.

SIRT1 is an NAD (+)-dependent histone deacetylase that is involved in biological processes, such as inflammation, stress response, and cell survival [37]. A previous study found that SIRT1 supports viral replication by modulating the human papillomavirus (HPV) replication complex [38], while another study further confirmed that the modulation of the acetylation of histones H1 and H4 by SIRT1 contributes to HPV viral replication and gene expression [39]. In HIV, SIRT1 specifically deacetylates the Tat protein, which is responsible for HIV reactivation, suggesting that SIRT1 can act as a cofactor for Tat-mediated HIV promoter activation [40]. Furthermore, SIRT1 induces hepatitis B virus (HBV) core promoter activity by targeting AP-1 and assists HBV replication in hepatocytes [41]. In this study, it was originally found that SIRT1 expression is elevated in RV replication, and that the downregulation of SIRT1 expression restrains autophagy and RV replication during RV infection. Furthermore, it was found that elevation of SIRT1 reverses the repressive effect caused by elevated miR-194-3p expression. This confirm that SIRT1 functions as a downstream molecule of miR-194-3p by regulating the progression of RV infection. Overall, these results suggest that SIRT1 inhibitors are very promising for the treatment of RV infections [42].

It has been reported that p53 is a tumor suppressor that plays an important role in regulating cell growth, proliferation, cell cycle progression, apoptosis, and immune responses [43]. A previous study showed that p53 acetylation is crucial for p53-mediated antiviral responses [20]. p53 is the first non-histone deacetylation target of SIRT1 and the SIRT1-p53 axis plays a crucial role in a variety of cellular processes [44]. Therefore, in this study, it was further explored whether RV infection affects the acetylation of p53 caused by SIRT1. It was found that elevated miR-194-3p expression or the inhibition of SIRT1 expression restrained the acetylation of p53, while downregulation of miR-194-3p expression promotes the acetylation of p53 by upregulating SIRT1. In addition, p53 deacetylation is also associated with autophagy. For example, p53 deacetylation alleviates sepsis-induced acute kidney injury by promoting autophagy [45], while p53 acetylation induces the apoptosis and autophagy of endometrial cancer cells [46]. However, it remains to be verified whether p53 acetylation has an effect on autophagy in RV-infected cells, and further investigations will be carried out in the future. Overall, the results of this study suggest that the miR-194-3p/SIRT1/p53 axis performs a crucial role for autophagy and RV replication during RV infection.

Although miR-194-3p was downregulated *in vivo and in vitro* in the RV-infected models, its suitability as a diagnostic biomarker for RV infection needs to be verified through the further detection of its expression in the serum of RV patients using multi-cohort analysis. Meanwhile, autophagy is a cellular response activated by many pathogens, and the effect of other RV strains, on the activation of autophagy and its latent mechanism still needs to be further explored to help refine the specific underlying mechanisms of miR-194-3p in RV replication.

## Conclusions

In conclusion, our present study proposes a novel regulatory mechanism of RV replication whereby miR-194-3p restrains RV replication by targeting SIRT1 to regulate p53 acetylation and to regulate autophagy. The results of this study offer novel insights into RV replication and a new target for the development of anti-RV drugs.

## Data Availability

The original contributions presented in the study are included in the article/supplementary material, further inquiries can be directed to the corresponding author/s.
